# (2*S*)-2-(4-Ethyl-2,3-dioxopiperazine-1-carboxamido)-2-(4-hy­droxy­phen­yl)acetic acid

**DOI:** 10.1107/S1600536810025262

**Published:** 2010-07-03

**Authors:** Qian Wang, Ling Hu, Jian-Ping Ma, Dian-Shun Guo

**Affiliations:** a Department of Chemistry, Shandong Normal University, Jinan 250014, People’s Republic of China

## Abstract

There are two mol­ecules in the asymmetric unit of the title compound, C_15_H_17_N_3_O_6_. The 2,3-dioxopiperazine ring adopts a half-chair conformation with torsion angles of −7.6 (4) and 35.1 (4)° in one mol­ecule, and 5.3 (4) and 45.4 (4)° in the other mol­ecule. In the crystal structure, the carb­oxy groups are involved in classical inversion-related O—H⋯O hydrogen bonds, which link the mol­ecules into centrosymmetric dimers. These dimers are further linked by inter­molecular O—H⋯O and C—H⋯O hydrogen bonds. Each independent mol­ecule also exhibits an intra­molecular N—H⋯O hydrogen bond. The H atoms of the carb­oxy groups are disordered over two positions, with refined site-occupancy factors of 0.5.

## Related literature

For general background to cefoperazone, a third generation cephalosporin anti­biotic, and HO-EPCP [(2*R*)-2-(4-ethyl-2,3-dioxopiperazine-1-carboxamido)-2-(4-hydroxyphenyl)acetic acid], which has been investigated extensively as a key inter­mediate for the synthesis of cefoperazone, see: Spyker *et al.* (1985 ▶); Chen *et al.* (2009 ▶); Murakami *et al.* (1981 ▶); Albrecht *et al.* (1991 ▶). For the synthesis of the (2*S*)-enantiomer of (HO-EPCP), see: De Lorenzi *et al.* (2001 ▶). For a related structure, see: Lenstra *et al.* (1998 ▶). For disordered carb­oxy dimers, see: Leiserowitz (1976 ▶); Feeder & Jones (1996 ▶). For helical chains, see: Adachi *et al.* (2001 ▶); Xu *et al.*(2003 ▶); Enamullah *et al.* (2006 ▶). For hydrogen-bond motifs, see: Bernstein *et al.* (1995 ▶). For the synthesis, see: Saikawa *et al.* (1978 ▶).
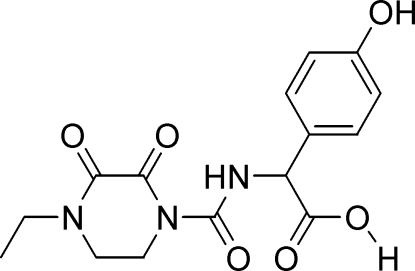


## Experimental

### 

#### Crystal data

C_15_H_17_N_3_O_6_
*M* *_r_* = 335.32
Orthorhombic, 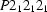

*a* = 11.5899 (19) Å
*b* = 13.038 (2) Å
*c* = 20.794 (3) Å
*V* = 3142.2 (9) Å^3^
*Z* = 8
Mo *K*α radiation
μ = 0.11 mm^−1^
*T* = 298 K
0.51 × 0.15 × 0.09 mm

#### Data collection

Bruker SMART CCD area-detector diffractometer
16467 measured reflections
3237 independent reflections
2722 reflections with *I* > 2σ(*I*)
*R*_int_ = 0.040

#### Refinement

*R*[*F* ^2^ > 2σ(*F* ^2^)] = 0.044
*wR*(*F* ^2^) = 0.107
*S* = 1.06
3237 reflections
449 parameters
16 restraints
H atoms treated by a mixture of independent and constrained refinement
Δρ_max_ = 0.18 e Å^−3^
Δρ_min_ = −0.16 e Å^−3^

### 

Data collection: *SMART* (Bruker, 1999 ▶); cell refinement: *SAINT* (Bruker, 1999 ▶); data reduction: *SAINT*; program(s) used to solve structure: *SHELXS97* (Sheldrick, 2008 ▶); program(s) used to refine structure: *SHELXL97* (Sheldrick, 2008 ▶); molecular graphics: *SHELXTL* (Sheldrick, 2008 ▶); software used to prepare material for publication: *SHELXTL*.

## Supplementary Material

Crystal structure: contains datablocks I, global. DOI: 10.1107/S1600536810025262/lx2155sup1.cif

Structure factors: contains datablocks I. DOI: 10.1107/S1600536810025262/lx2155Isup2.hkl

Additional supplementary materials:  crystallographic information; 3D view; checkCIF report

## Figures and Tables

**Table 1:** Hydrogen-bond geometry (Å, °)

*D*—H⋯*A*	*D*—H	H⋯*A*	*D*⋯*A*	*D*—H⋯*A*
O11—H11*O*⋯O5	0.86 (3)	1.80 (4)	2.617 (3)	159 (9)
O10—H10*O*⋯O4	0.86 (3)	1.82 (3)	2.678 (3)	171 (9)
O5—H5*O*⋯O11	0.86 (3)	1.76 (3)	2.617 (3)	171 (8)
O4—H4*O*⋯O10	0.86 (3)	1.88 (5)	2.678 (3)	154 (9)
N6—H6*D*⋯O8	0.86	1.98	2.637 (3)	132
N3—H3⋯O2	0.86	1.97	2.636 (3)	133
O6—H6⋯O1^i^	0.82	1.87	2.682 (3)	174
C12—H12⋯O2^i^	0.93	2.52	3.438 (4)	169
O12—H12*A*⋯O7^ii^	0.82	1.88	2.692 (3)	172
C27—H27⋯O8^ii^	0.93	2.50	3.378 (4)	158

Symmetry codes: (i) 
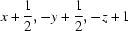
; (ii) 
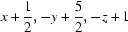
.

**Fig. 1.**
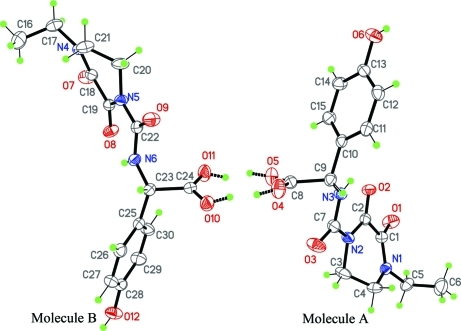
A view of the title compound, showing 30% probability displacement ellipsoids. The disordered moieties (dotted lines) are shown.

**Fig. 2.**
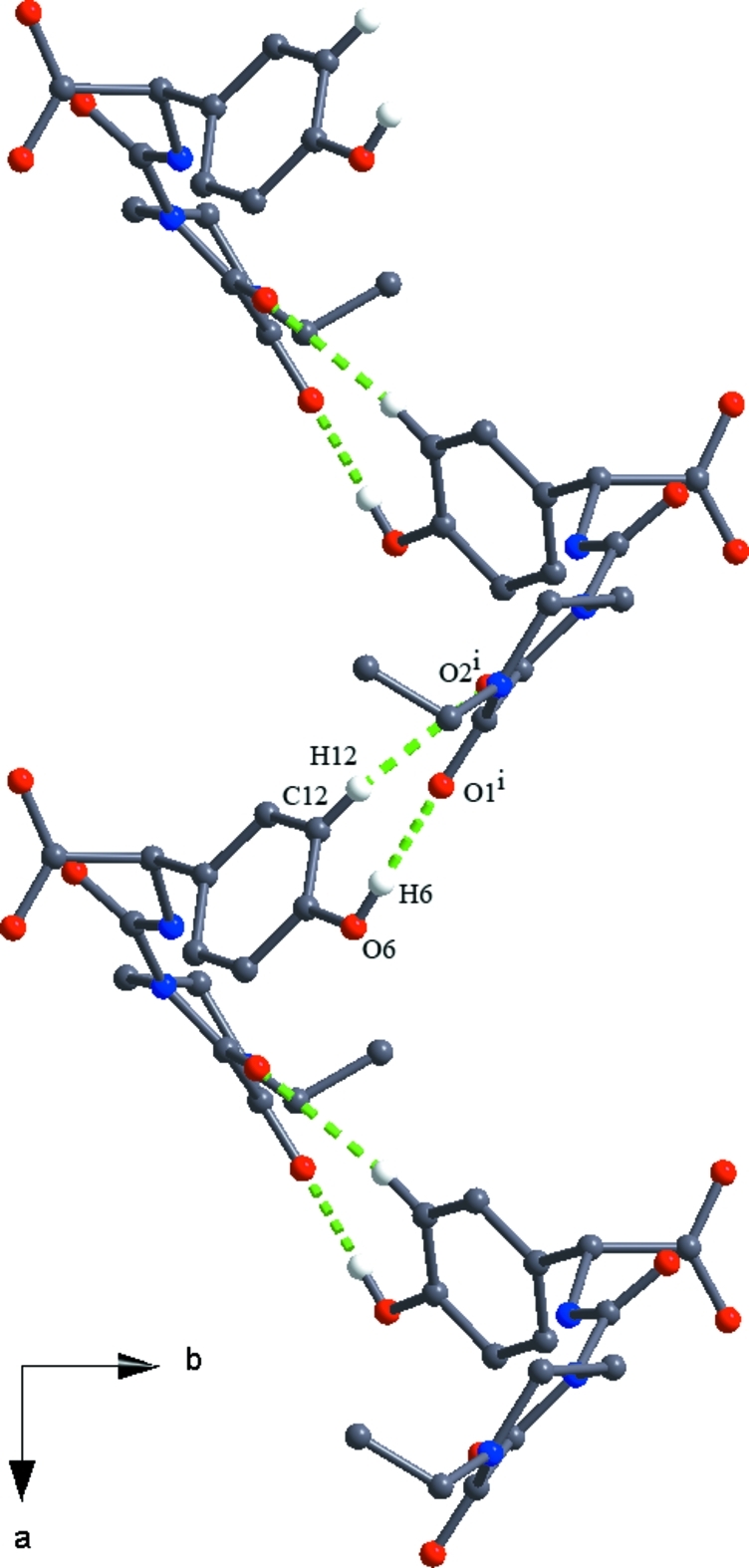
The hydrogen-bonded one-dimensional helix chain of the molecule A with an R22(9) ring motif viewed along the c axis. The disordered moieties and some hydrogen atoms are omitted for clarity. [Symmetry code: (i) x + 1/2, - y + 1/2, - z + 1].

**Fig. 3.**
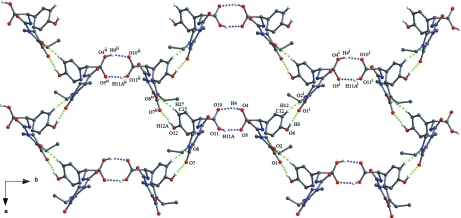
The two-dimensional network of the title compound with R22(8) and R22(9) ring motifs viewed along the c axis. The disordered moieties and some hydrogen atoms are omitted for clarity. [Symmetry codes: (i) x + 1/2, - y + 1/2, - z + 1; (ii) x + 1/2, - y+ 5/2, - z + 1].

## supplementary crystallographic information

### Comment

Among enantiomers of 2-(4-ethyl-3-dioxo-1-piperazinyl)carboxamido]-2-(4-hydroxyphenyl)acetic acid, its (2*R*)-enantiomer (HO-EPCP) has been extensively investigated as a key intermediate for the synthesis of cefoperazone (Murakami *et al.*, 1981; Albrecht *et al.*, 1991), which is a third generation cephalosporin antibiotic (Spyker *et al.*, 1985; Chen *et al.*, 2009), whereas its (2*S*)-enantiomer was presented only one time as a chiral compound of pharmaceutical interest evaluated for enantiorecognition (De Lorenzi *et al.*, 2001). Now we report the crystal structure of the title compound which crystallizes with two unique molecules, denoted as A & B, in the asymmetric unit (Fig. 1).

The two molecules (A and B) are linked into a dimer through classical inversion-related O—H···O hydrogen bonds (Table 1) between the carboxy groups of A and B, where the H atoms of the carboxy groups are disordered over two positions, with refined site-occupancy factors of 0.5 and 0.5. The disordered model for the carboxy group may be ascribed to the requirement of intermolecular forces (Leiserowitz, 1976; Feeder *et al.*, 1996). Both molecules possess similar geometric parameters except for the slight differences in some bond lenghths and angles. The 2,3-dioxopiperazine ring adopts a half-chair conformation with torsion angles of -7.6 (4) and 35.1 (4)° in A, while 5.3 (4) and 45.4 (4)° in B, similar to those of -3.3 (4) and 38.6 (4)° reported previously for the related molecule 2,3-diketopiperazine (Lenstra *et al.*, 1998). The dihedral angle between the two benzene rings belonging to A and B in the dimer is 47.32 (10)°. The intramolecular N3—H3···O2 and N6—H6*D*···O8 hydrogen bonds exist (Table 1) and create an *R*(6) ring motif (Bernstein *et al.*, 1995) in either molecule.

The packing of the title compound is obviously stabilized by the intermolecular O—H···O and C—H···O hydrogen bonds. For the molecule A, an infinite one-dimensional helix chain (Adachi *et al.* 2001; Xu *et al. *2003) is formed by a combination of the intermolecular O—H···O and C—H···O hydrogen bonds (Fig. 2), locally giving an *R*_2_^2^(9) ring motif (Bernstein *et al.*, 1995). This motif arises from atoms O6—H6 and C12—H12 in the molecule at (*x*, *y*, *z*), which act as hydrogen-bond donors, to atoms O1 and O2 in the neighboring molecule at (*x* + 1/2, -*y* + 1/2, -*z + *1). For the molecule B, the same infinite one-dimensional helix chain is generated through a combination of the intermolecular O12—H12*A*···O7^ii^ and C27—H27···O8^ii^ [symmetry code: (ii) *x* + 1/2, -*y* + 5/2, -*z + *1] hydrogen bonds. Finally, the helix chains of both molecules are alternatively linked by the interchain O—H···O hydrogen bonds to produce a two-dimensional network (Fig. 3) in the *ab* plane. Such helix chains of the title molecule may be significant in simulating of the life system.

### Experimental

The title compound was obtained as a white solid in 80% yield by a similar method used for the synthesis of its (2*R*)-enantiomer (Saikawa *et al.*, 1978). ^1^H NMR (300 MHz, DMSO-*d*_6_): *δ* 9.68 (d, 1H, *J* = 6.42 Hz), 9.58 (s, 1H), 7.18 (d, 2H, *J* = 8.27 Hz), 6.77 (d, 2H, *J* = 8.27 Hz), 5.20 (d, 1H, *J* = 6.44 Hz), 3.90 (br, 2H), 3.56 (br, 2H), 3.40 (m, 2H), 1.08 (t, 3H, *J* = 7.06 Hz). Single crystals of the title compound suitable for *X*-ray diffraction analysis were obtained by slow cooling of a hot solution in H_2_O.

### Refinement

Hydrogen atoms attached to refined atoms were placed in geometrically idealized positions and refined using a riding model, with C—H = 0.93, 0.97 and 0.96 Å for aromatic, methylene and methyl H, respectively, and *U*_iso_(H) = 1.5Ueq(C) for methyl H, and *U*_iso_(H) =1.2Ueq(C) for all other H atoms. The H atoms (H4*O*, H5*O*, H11*O*, and H10*O*) of carboxy groups were located in the different map and refined isotropically subject to an O—H = 0.85 (10) Å distance over two orientations in a 50/50 ratio. These C—O bonds were restrained to be the same within a standard deviation of 0.01 Å, total 16 restrains were used to model the two carboxy groups. In the absence of significant anomalous scattering effects, measured Friedel pairs were merged.

### Crystal data

**Table:**

C_15_H_17_N_3_O_6_	*F*(000) = 1408
*M**_r_* = 335.32	*D*_x_ = 1.418 Mg m^−^^3^
Orthorhombic, *P*2_1_2_1_2_1_	Mo *K*α radiation, λ = 0.71073 Å
Hall symbol: P 2ac 2ab	Cell parameters from 3149 reflections
*a* = 11.5899 (19) Å	θ = 2.5–22.5°
*b* = 13.038 (2) Å	µ = 0.11 mm^−^^1^
*c* = 20.794 (3) Å	*T* = 298 K
*V* = 3142.2 (9) Å^3^	Bar, colourless
*Z* = 8	0.51 × 0.15 × 0.09 mm

### Data collection

**Table:**

Bruker SMART CCD area-detector diffractometer	2722 reflections with *I* > 2σ(*I*)
Radiation source: fine-focus sealed tube	*R*_int_ = 0.040
graphite	θ_max_ = 25.4°, θ_min_ = 1.8°
Detector resolution: 10.0 pixels mm^-1^	*h* = −13→13
phi and ω scans	*k* = −15→15
16467 measured reflections	*l* = −18→25
3237 independent reflections	

### Refinement

**Table:**

Refinement on *F*^2^	Primary atom site location: structure-invariant direct methods
Least-squares matrix: full	Secondary atom site location: difference Fourier map
*R*[*F*^2^ > 2σ(*F*^2^)] = 0.044	Hydrogen site location: difference Fourier map
*wR*(*F*^2^) = 0.107	H atoms treated by a mixture of independent and constrained refinement
*S* = 1.06	*w* = 1/[σ^2^(*F*_o_^2^) + (0.0613*P*)^2^ + 0.0947*P*] where *P* = (*F*_o_^2^ + 2*F*_c_^2^)/3
3237 reflections	(Δ/σ)_max_ < 0.001
449 parameters	Δρ_max_ = 0.18 e Å^−^^3^
16 restraints	Δρ_min_ = −0.16 e Å^−^^3^

### Special details

**Table:**

Geometry. All e.s.d.'s (except the e.s.d. in the dihedral angle between two l.s. planes) are estimated using the full covariance matrix. The cell e.s.d.'s are taken into account individually in the estimation of e.s.d.'s in distances, angles and torsion angles; correlations between e.s.d.'s in cell parameters are only used when they are defined by crystal symmetry. An approximate (isotropic) treatment of cell e.s.d.'s is used for estimating e.s.d.'s involving l.s. planes.
Refinement. Refinement of *F*^2^ against ALL reflections. The weighted *R*-factor *wR* and goodness of fit *S* are based on *F*^2^, conventional *R*-factors *R* are based on *F*, with *F* set to zero for negative *F*^2^. The threshold expression of *F*^2^ > σ(*F*^2^) is used only for calculating *R*-factors(gt) *etc*. and is not relevant to the choice of reflections for refinement. *R*-factors based on *F*^2^ are statistically about twice as large as those based on *F*, and *R*- factors based on ALL data will be even larger.

### Fractional atomic coordinates and isotropic or equivalent isotropic displacement parameters (Å^2^)

**Table:**

	*x*	*y*	*z*	*U*_iso_*/*U*_eq_	Occ. (<1)
O5	0.6862 (2)	0.66654 (17)	0.51841 (12)	0.0584 (6)	
C1	0.4645 (3)	0.3773 (2)	0.73859 (14)	0.0421 (7)	
C2	0.5301 (3)	0.4183 (2)	0.67963 (14)	0.0379 (7)	
C3	0.6229 (4)	0.5339 (3)	0.75568 (14)	0.0608 (10)	
H3A	0.5614	0.5831	0.7630	0.073*	
H3B	0.6960	0.5696	0.7598	0.073*	
C4	0.6161 (3)	0.4511 (3)	0.80428 (14)	0.0515 (8)	
H4A	0.6815	0.4052	0.7994	0.062*	
H4B	0.6191	0.4805	0.8471	0.062*	
C5	0.4652 (3)	0.3361 (3)	0.85232 (14)	0.0534 (9)	
H5A	0.3841	0.3206	0.8457	0.064*	
H5B	0.4717	0.3781	0.8906	0.064*	
C6	0.5296 (4)	0.2396 (3)	0.86191 (19)	0.0841 (13)	
H6A	0.4973	0.2028	0.8976	0.126*	
H6B	0.6091	0.2549	0.8705	0.126*	
H6C	0.5242	0.1983	0.8238	0.126*	
C7	0.6935 (3)	0.5273 (2)	0.64418 (14)	0.0468 (8)	
C8	0.78013 (16)	0.6197 (2)	0.52363 (14)	0.0420 (7)	
C9	0.7784 (3)	0.5059 (2)	0.53998 (13)	0.0417 (7)	
H9	0.8536	0.4865	0.5578	0.050*	
C10	0.7571 (3)	0.4445 (2)	0.47940 (13)	0.0392 (7)	
C11	0.8371 (3)	0.3733 (2)	0.45867 (14)	0.0445 (8)	
H11	0.9052	0.3646	0.4817	0.053*	
C12	0.8176 (3)	0.3146 (2)	0.40408 (15)	0.0474 (8)	
H12	0.8723	0.2671	0.3905	0.057*	
C13	0.7164 (3)	0.3271 (2)	0.37011 (14)	0.0435 (8)	
C14	0.6354 (3)	0.3972 (3)	0.39031 (15)	0.0507 (8)	
H14	0.5671	0.4058	0.3674	0.061*	
C15	0.6563 (3)	0.4549 (2)	0.44491 (15)	0.0480 (8)	
H15	0.6011	0.5018	0.4587	0.058*	
C16	0.4769 (4)	1.2845 (3)	0.1574 (2)	0.0929 (15)	
H16A	0.4413	1.3066	0.1967	0.139*	
H16B	0.4416	1.3192	0.1218	0.139*	
H16C	0.5577	1.3003	0.1585	0.139*	
C17	0.4611 (3)	1.1708 (3)	0.14984 (15)	0.0552 (9)	
H17A	0.3794	1.1548	0.1495	0.066*	
H17B	0.4935	1.1492	0.1090	0.066*	
C18	0.4657 (3)	1.1091 (2)	0.25917 (14)	0.0415 (7)	
C19	0.5391 (3)	1.0779 (2)	0.31764 (13)	0.0379 (7)	
C20	0.6653 (3)	0.9982 (3)	0.23883 (13)	0.0564 (9)	
H20A	0.6198	0.9381	0.2282	0.068*	
H20B	0.7462	0.9806	0.2344	0.068*	
C21	0.6363 (3)	1.0832 (3)	0.19387 (14)	0.0535 (9)	
H21A	0.6867	1.1412	0.2020	0.064*	
H21B	0.6484	1.0608	0.1499	0.064*	
C22	0.7251 (3)	1.0017 (2)	0.35317 (14)	0.0413 (7)	
C23	0.7895 (3)	1.0151 (2)	0.46270 (13)	0.0400 (7)	
H23	0.8671	1.0322	0.4474	0.048*	
C24	0.78669 (17)	0.9022 (2)	0.48071 (13)	0.0403 (7)	
C25	0.7646 (3)	1.0775 (2)	0.52265 (13)	0.0380 (7)	
C26	0.8464 (3)	1.1431 (2)	0.54761 (15)	0.0450 (7)	
H26	0.9170	1.1500	0.5268	0.054*	
C27	0.8258 (3)	1.1987 (2)	0.60304 (14)	0.0484 (8)	
H27	0.8823	1.2423	0.6193	0.058*	
C28	0.7214 (3)	1.1894 (2)	0.63420 (14)	0.0441 (8)	
C29	0.6382 (3)	1.1250 (3)	0.60949 (15)	0.0540 (9)	
H29	0.5673	1.1188	0.6301	0.065*	
C30	0.6598 (3)	1.0695 (2)	0.55422 (15)	0.0494 (8)	
H30	0.6032	1.0262	0.5380	0.059*	
N1	0.5094 (2)	0.39370 (19)	0.79653 (11)	0.0421 (6)	
N2	0.6125 (2)	0.49079 (18)	0.69051 (11)	0.0448 (6)	
N3	0.6901 (2)	0.48256 (18)	0.58659 (10)	0.0440 (6)	
H3	0.6357	0.4402	0.5773	0.053*	
N4	0.5174 (2)	1.11428 (19)	0.20220 (11)	0.0412 (6)	
N5	0.6415 (2)	1.02945 (18)	0.30565 (10)	0.0402 (6)	
N6	0.7073 (2)	1.03845 (17)	0.41218 (10)	0.0421 (6)	
H6D	0.6477	1.0756	0.4202	0.051*	
O1	0.3770 (2)	0.3277 (2)	0.72961 (11)	0.0663 (7)	
O2	0.50699 (18)	0.38196 (17)	0.62759 (9)	0.0482 (6)	
O3	0.7594 (2)	0.59548 (19)	0.65771 (11)	0.0759 (9)	
O4	0.8770 (2)	0.65925 (18)	0.51346 (13)	0.0615 (7)	
H4O	0.900 (8)	0.722 (3)	0.509 (4)	0.092*	0.50
H5O	0.687 (9)	0.726 (3)	0.500 (4)	0.092*	0.50
O6	0.6918 (2)	0.27188 (18)	0.31598 (10)	0.0590 (6)	
H6	0.7493	0.2401	0.3046	0.088*	
O7	0.3658 (2)	1.1346 (2)	0.26865 (11)	0.0609 (7)	
O8	0.50410 (18)	1.10077 (18)	0.37060 (9)	0.0507 (6)	
O9	0.8068 (2)	0.94970 (17)	0.33806 (10)	0.0625 (7)	
O10	0.8777 (2)	0.86393 (17)	0.50285 (12)	0.0532 (6)	
H10O	0.881 (7)	0.798 (2)	0.503 (4)	0.080*	0.50
O11	0.6930 (2)	0.85492 (17)	0.47513 (12)	0.0604 (7)	
H11O	0.679 (8)	0.800 (4)	0.496 (4)	0.091*	0.50
O12	0.6966 (2)	1.24064 (19)	0.68955 (10)	0.0615 (7)	
H12A	0.7521	1.2758	0.7000	0.092*	

### Atomic displacement parameters (Å^2^)

**Table:**

	*U*^11^	*U*^22^	*U*^33^	*U*^12^	*U*^13^	*U*^23^
O5	0.0529 (15)	0.0450 (13)	0.0772 (16)	0.0050 (12)	0.0103 (13)	0.0107 (12)
C1	0.0428 (19)	0.0438 (17)	0.0398 (17)	−0.0002 (16)	0.0001 (15)	−0.0009 (13)
C2	0.0387 (17)	0.0364 (15)	0.0386 (17)	0.0030 (14)	0.0005 (14)	0.0016 (13)
C3	0.085 (3)	0.059 (2)	0.0387 (18)	−0.026 (2)	0.0137 (18)	−0.0173 (16)
C4	0.057 (2)	0.066 (2)	0.0320 (16)	−0.0083 (18)	0.0017 (15)	−0.0093 (15)
C5	0.057 (2)	0.064 (2)	0.0388 (17)	0.0020 (18)	0.0073 (16)	0.0106 (15)
C6	0.115 (4)	0.065 (3)	0.072 (3)	0.010 (3)	0.007 (3)	0.021 (2)
C7	0.060 (2)	0.0418 (16)	0.0389 (17)	−0.0105 (17)	0.0088 (15)	0.0007 (14)
C8	0.046 (2)	0.0422 (16)	0.0375 (16)	−0.0016 (16)	0.0079 (15)	0.0004 (13)
C9	0.0481 (19)	0.0394 (15)	0.0375 (16)	0.0039 (15)	0.0048 (14)	0.0043 (12)
C10	0.0469 (19)	0.0338 (14)	0.0370 (15)	0.0008 (13)	0.0073 (14)	0.0068 (12)
C11	0.0459 (19)	0.0422 (16)	0.0454 (17)	0.0058 (15)	0.0025 (15)	0.0069 (14)
C12	0.051 (2)	0.0400 (15)	0.0511 (19)	0.0093 (15)	0.0143 (16)	0.0010 (14)
C13	0.055 (2)	0.0370 (15)	0.0383 (16)	−0.0053 (15)	0.0132 (15)	−0.0008 (13)
C14	0.051 (2)	0.0588 (19)	0.0428 (18)	0.0054 (18)	0.0011 (15)	−0.0040 (16)
C15	0.050 (2)	0.0492 (18)	0.0447 (18)	0.0147 (16)	0.0060 (16)	−0.0026 (15)
C16	0.126 (4)	0.071 (3)	0.082 (3)	0.003 (3)	−0.029 (3)	0.025 (2)
C17	0.046 (2)	0.081 (2)	0.0387 (18)	0.0045 (19)	−0.0083 (16)	0.0017 (17)
C18	0.044 (2)	0.0415 (17)	0.0391 (17)	−0.0024 (15)	−0.0020 (15)	−0.0052 (13)
C19	0.0425 (18)	0.0359 (15)	0.0353 (16)	−0.0011 (14)	0.0030 (14)	−0.0010 (12)
C20	0.064 (2)	0.069 (2)	0.0363 (17)	0.026 (2)	−0.0042 (16)	−0.0180 (16)
C21	0.047 (2)	0.080 (2)	0.0333 (17)	0.0110 (19)	0.0011 (14)	−0.0053 (16)
C22	0.0510 (19)	0.0342 (14)	0.0387 (16)	0.0073 (15)	−0.0022 (15)	0.0003 (12)
C23	0.0442 (18)	0.0400 (15)	0.0357 (15)	−0.0017 (14)	−0.0033 (13)	0.0050 (12)
C24	0.0473 (19)	0.0417 (15)	0.0319 (15)	−0.0015 (16)	−0.0054 (14)	−0.0003 (12)
C25	0.0423 (18)	0.0354 (14)	0.0364 (15)	0.0009 (13)	−0.0058 (14)	0.0050 (12)
C26	0.0423 (19)	0.0475 (17)	0.0453 (17)	−0.0079 (15)	0.0012 (14)	0.0069 (14)
C27	0.051 (2)	0.0456 (17)	0.0486 (18)	−0.0088 (16)	−0.0108 (16)	−0.0060 (14)
C28	0.049 (2)	0.0426 (15)	0.0408 (17)	0.0048 (16)	−0.0101 (15)	0.0007 (13)
C29	0.049 (2)	0.065 (2)	0.0489 (19)	−0.0050 (19)	0.0057 (16)	−0.0076 (16)
C30	0.047 (2)	0.0521 (19)	0.0492 (19)	−0.0146 (16)	−0.0025 (16)	−0.0068 (15)
N1	0.0449 (16)	0.0437 (13)	0.0377 (13)	0.0032 (12)	0.0022 (12)	0.0041 (11)
N2	0.0634 (18)	0.0390 (13)	0.0320 (13)	−0.0106 (13)	0.0090 (12)	−0.0028 (11)
N3	0.0565 (17)	0.0417 (13)	0.0339 (13)	−0.0120 (13)	0.0062 (12)	0.0009 (11)
N4	0.0406 (15)	0.0501 (14)	0.0330 (13)	0.0015 (12)	−0.0020 (11)	−0.0019 (11)
N5	0.0478 (16)	0.0409 (13)	0.0318 (12)	0.0108 (12)	−0.0036 (11)	−0.0057 (10)
N6	0.0526 (17)	0.0398 (13)	0.0339 (13)	0.0080 (13)	−0.0028 (12)	0.0020 (11)
O1	0.0525 (16)	0.0900 (18)	0.0564 (15)	−0.0224 (15)	−0.0003 (12)	0.0063 (14)
O2	0.0522 (14)	0.0569 (13)	0.0355 (12)	−0.0080 (11)	−0.0067 (10)	−0.0026 (10)
O3	0.099 (2)	0.0766 (16)	0.0520 (14)	−0.0526 (17)	0.0165 (14)	−0.0137 (13)
O4	0.0525 (16)	0.0522 (14)	0.0799 (17)	−0.0074 (13)	0.0115 (13)	0.0142 (13)
O6	0.0652 (16)	0.0586 (14)	0.0530 (14)	−0.0088 (13)	0.0116 (12)	−0.0176 (11)
O7	0.0371 (14)	0.0910 (18)	0.0545 (14)	0.0106 (13)	0.0005 (11)	−0.0080 (13)
O8	0.0486 (14)	0.0694 (14)	0.0341 (12)	0.0074 (12)	0.0080 (10)	0.0019 (10)
O9	0.0718 (17)	0.0650 (15)	0.0508 (13)	0.0346 (14)	−0.0129 (12)	−0.0133 (11)
O10	0.0516 (14)	0.0462 (12)	0.0619 (14)	0.0026 (12)	−0.0150 (11)	0.0048 (11)
O11	0.0536 (15)	0.0461 (12)	0.0816 (18)	−0.0110 (12)	−0.0200 (14)	0.0137 (12)
O12	0.0635 (17)	0.0705 (16)	0.0504 (13)	0.0077 (14)	−0.0068 (12)	−0.0234 (12)

### Geometric parameters (Å, °)

**Table:**

O5—C8	1.2534 (17)	C16—H16C	0.9600
O5—O10	3.414 (3)	C17—N4	1.468 (4)
O5—H5O	0.86 (3)	C17—H17A	0.9700
C1—O1	1.217 (4)	C17—H17B	0.9700
C1—N1	1.330 (4)	C18—O7	1.221 (4)
C1—C2	1.539 (4)	C18—N4	1.329 (4)
C2—O2	1.211 (3)	C18—C19	1.539 (4)
C2—N2	1.362 (4)	C19—O8	1.211 (3)
C3—N2	1.472 (4)	C19—N5	1.367 (4)
C3—C4	1.482 (5)	C20—N5	1.474 (3)
C3—H3A	0.9700	C20—C21	1.488 (4)
C3—H3B	0.9700	C20—H20A	0.9700
C4—N1	1.454 (4)	C20—H20B	0.9700
C4—H4A	0.9700	C21—N4	1.447 (4)
C4—H4B	0.9700	C21—H21A	0.9700
C5—N1	1.474 (4)	C21—H21B	0.9700
C5—C6	1.476 (5)	C22—O9	1.206 (4)
C5—H5A	0.9700	C22—N6	1.333 (3)
C5—H5B	0.9700	C22—N5	1.431 (4)
C6—H6A	0.9600	C23—N6	1.450 (4)
C6—H6B	0.9600	C23—C25	1.516 (4)
C6—H6C	0.9600	C23—C24	1.520 (4)
C7—O3	1.206 (4)	C23—H23	0.9800
C7—N3	1.332 (4)	C24—O10	1.2537 (17)
C7—N2	1.427 (4)	C24—O11	1.2538 (17)
C8—O4	1.2538 (17)	C25—C26	1.378 (4)
C8—C9	1.523 (4)	C25—C30	1.384 (4)
C9—N3	1.442 (4)	C26—C27	1.383 (4)
C9—C10	1.513 (4)	C26—H26	0.9300
C9—H9	0.9800	C27—C28	1.378 (5)
C10—C15	1.378 (4)	C27—H27	0.9300
C10—C11	1.381 (4)	C28—O12	1.362 (3)
C11—C12	1.388 (4)	C28—C29	1.379 (4)
C11—H11	0.9300	C29—C30	1.381 (4)
C12—C13	1.379 (5)	C29—H29	0.9300
C12—H12	0.9300	C30—H30	0.9300
C13—O6	1.367 (3)	N3—H3	0.8600
C13—C14	1.376 (4)	N6—H6D	0.8600
C14—C15	1.383 (4)	O4—H4O	0.86 (3)
C14—H14	0.9300	O6—H6	0.8200
C15—H15	0.9300	O10—H10O	0.86 (3)
C16—C17	1.501 (6)	O11—H11O	0.86 (3)
C16—H16A	0.9600	O12—H12A	0.8200
C16—H16B	0.9600		
			
C8—O5—O10	79.07 (15)	H17A—C17—H17B	108.0
C8—O5—H5O	117 (7)	O7—C18—N4	123.9 (3)
O10—O5—H5O	43 (7)	O7—C18—C19	118.0 (3)
O1—C1—N1	123.4 (3)	N4—C18—C19	117.9 (3)
O1—C1—C2	118.3 (3)	O8—C19—N5	124.8 (3)
N1—C1—C2	118.2 (3)	O8—C19—C18	117.9 (3)
O2—C2—N2	125.1 (3)	N5—C19—C18	117.3 (2)
O2—C2—C1	117.8 (3)	N5—C20—C21	110.1 (3)
N2—C2—C1	117.1 (3)	N5—C20—H20A	109.6
N2—C3—C4	110.2 (3)	C21—C20—H20A	109.6
N2—C3—H3A	109.6	N5—C20—H20B	109.6
C4—C3—H3A	109.6	C21—C20—H20B	109.6
N2—C3—H3B	109.6	H20A—C20—H20B	108.1
C4—C3—H3B	109.6	N4—C21—C20	110.4 (3)
H3A—C3—H3B	108.1	N4—C21—H21A	109.6
N1—C4—C3	110.2 (3)	C20—C21—H21A	109.6
N1—C4—H4A	109.6	N4—C21—H21B	109.6
C3—C4—H4A	109.6	C20—C21—H21B	109.6
N1—C4—H4B	109.6	H21A—C21—H21B	108.1
C3—C4—H4B	109.6	O9—C22—N6	124.3 (3)
H4A—C4—H4B	108.1	O9—C22—N5	119.6 (3)
N1—C5—C6	111.4 (3)	N6—C22—N5	116.1 (3)
N1—C5—H5A	109.3	N6—C23—C25	111.0 (2)
C6—C5—H5A	109.3	N6—C23—C24	111.6 (2)
N1—C5—H5B	109.3	C25—C23—C24	108.3 (2)
C6—C5—H5B	109.3	N6—C23—H23	108.7
H5A—C5—H5B	108.0	C25—C23—H23	108.7
C5—C6—H6A	109.5	C24—C23—H23	108.7
C5—C6—H6B	109.5	O10—C24—O11	124.5 (3)
H6A—C6—H6B	109.5	O10—C24—C23	117.2 (2)
C5—C6—H6C	109.5	O11—C24—C23	118.2 (2)
H6A—C6—H6C	109.5	C26—C25—C30	118.2 (3)
H6B—C6—H6C	109.5	C26—C25—C23	120.7 (3)
O3—C7—N3	123.5 (3)	C30—C25—C23	121.1 (3)
O3—C7—N2	120.3 (3)	C25—C26—C27	121.3 (3)
N3—C7—N2	116.2 (3)	C25—C26—H26	119.3
O5—C8—O4	124.3 (3)	C27—C26—H26	119.3
O5—C8—C9	118.9 (2)	C28—C27—C26	119.8 (3)
O4—C8—C9	116.8 (2)	C28—C27—H27	120.1
N3—C9—C10	109.4 (2)	C26—C27—H27	120.1
N3—C9—C8	111.4 (2)	O12—C28—C27	122.7 (3)
C10—C9—C8	109.4 (2)	O12—C28—C29	117.8 (3)
N3—C9—H9	108.9	C27—C28—C29	119.5 (3)
C10—C9—H9	108.9	C28—C29—C30	120.1 (3)
C8—C9—H9	108.9	C28—C29—H29	119.9
C15—C10—C11	118.2 (3)	C30—C29—H29	119.9
C15—C10—C9	121.3 (3)	C29—C30—C25	121.0 (3)
C11—C10—C9	120.4 (3)	C29—C30—H30	119.5
C10—C11—C12	121.1 (3)	C25—C30—H30	119.5
C10—C11—H11	119.4	C1—N1—C4	121.1 (3)
C12—C11—H11	119.4	C1—N1—C5	119.7 (3)
C13—C12—C11	119.5 (3)	C4—N1—C5	118.1 (2)
C13—C12—H12	120.3	C2—N2—C7	125.5 (2)
C11—C12—H12	120.3	C2—N2—C3	118.4 (2)
O6—C13—C14	117.3 (3)	C7—N2—C3	116.1 (3)
O6—C13—C12	122.5 (3)	C7—N3—C9	119.4 (3)
C14—C13—C12	120.2 (3)	C7—N3—H3	120.3
C13—C14—C15	119.5 (3)	C9—N3—H3	120.3
C13—C14—H14	120.3	C18—N4—C21	121.4 (3)
C15—C14—H14	120.3	C18—N4—C17	119.1 (3)
C10—C15—C14	121.5 (3)	C21—N4—C17	118.4 (2)
C10—C15—H15	119.3	C19—N5—C22	125.4 (2)
C14—C15—H15	119.3	C19—N5—C20	117.5 (2)
C17—C16—H16A	109.5	C22—N5—C20	117.0 (2)
C17—C16—H16B	109.5	C22—N6—C23	119.3 (3)
H16A—C16—H16B	109.5	C22—N6—H6D	120.4
C17—C16—H16C	109.5	C23—N6—H6D	120.4
H16A—C16—H16C	109.5	C8—O4—H4O	133 (6)
H16B—C16—H16C	109.5	C13—O6—H6	109.5
N4—C17—C16	111.3 (3)	C24—O10—O5	77.75 (16)
N4—C17—H17A	109.4	C24—O10—H10O	116 (6)
C16—C17—H17A	109.4	O5—O10—H10O	44 (6)
N4—C17—H17B	109.4	C24—O11—H11O	122 (6)
C16—C17—H17B	109.4	C28—O12—H12A	109.5
			
O1—C1—C2—O2	−15.5 (4)	O1—C1—N1—C4	178.2 (3)
N1—C1—C2—O2	161.3 (3)	C2—C1—N1—C4	1.6 (4)
O1—C1—C2—N2	166.5 (3)	O1—C1—N1—C5	10.9 (5)
N1—C1—C2—N2	−16.8 (4)	C2—C1—N1—C5	−165.7 (3)
N2—C3—C4—N1	−56.5 (4)	C3—C4—N1—C1	35.1 (4)
O10—O5—C8—O4	−8.8 (3)	C3—C4—N1—C5	−157.4 (3)
O10—O5—C8—C9	174.5 (3)	C6—C5—N1—C1	90.2 (4)
O5—C8—C9—N3	−39.2 (4)	C6—C5—N1—C4	−77.4 (4)
O4—C8—C9—N3	143.9 (3)	O2—C2—N2—C7	−7.5 (5)
O5—C8—C9—C10	81.9 (3)	C1—C2—N2—C7	170.4 (3)
O4—C8—C9—C10	−95.0 (3)	O2—C2—N2—C3	174.5 (3)
N3—C9—C10—C15	59.9 (3)	C1—C2—N2—C3	−7.6 (4)
C8—C9—C10—C15	−62.4 (3)	O3—C7—N2—C2	176.3 (3)
N3—C9—C10—C11	−117.6 (3)	N3—C7—N2—C2	−3.0 (4)
C8—C9—C10—C11	120.1 (3)	O3—C7—N2—C3	−5.7 (5)
C15—C10—C11—C12	0.8 (4)	N3—C7—N2—C3	175.0 (3)
C9—C10—C11—C12	178.4 (3)	C4—C3—N2—C2	44.0 (4)
C10—C11—C12—C13	−0.3 (4)	C4—C3—N2—C7	−134.1 (3)
C11—C12—C13—O6	180.0 (3)	O3—C7—N3—C9	8.6 (5)
C11—C12—C13—C14	−0.1 (4)	N2—C7—N3—C9	−172.2 (3)
O6—C13—C14—C15	179.9 (3)	C10—C9—N3—C7	178.9 (3)
C12—C13—C14—C15	0.0 (5)	C8—C9—N3—C7	−60.1 (3)
C11—C10—C15—C14	−1.0 (4)	O7—C18—N4—C21	179.8 (3)
C9—C10—C15—C14	−178.5 (3)	C19—C18—N4—C21	5.3 (4)
C13—C14—C15—C10	0.6 (5)	O7—C18—N4—C17	11.8 (5)
O7—C18—C19—O8	−15.6 (4)	C19—C18—N4—C17	−162.6 (3)
N4—C18—C19—O8	159.2 (3)	C20—C21—N4—C18	32.0 (4)
O7—C18—C19—N5	166.6 (3)	C20—C21—N4—C17	−159.9 (3)
N4—C18—C19—N5	−18.6 (4)	C16—C17—N4—C18	78.3 (4)
N5—C20—C21—N4	−56.3 (4)	C16—C17—N4—C21	−90.0 (4)
N6—C23—C24—O10	152.5 (3)	O8—C19—N5—C22	−2.6 (5)
C25—C23—C24—O10	−85.1 (3)	C18—C19—N5—C22	175.0 (3)
N6—C23—C24—O11	−30.5 (4)	O8—C19—N5—C20	174.1 (3)
C25—C23—C24—O11	91.8 (3)	C18—C19—N5—C20	−8.4 (4)
N6—C23—C25—C26	−120.9 (3)	O9—C22—N5—C19	172.3 (3)
C24—C23—C25—C26	116.4 (3)	N6—C22—N5—C19	−9.2 (4)
N6—C23—C25—C30	59.8 (3)	O9—C22—N5—C20	−4.4 (4)
C24—C23—C25—C30	−62.9 (3)	N6—C22—N5—C20	174.2 (3)
C30—C25—C26—C27	0.7 (4)	C21—C20—N5—C19	45.4 (4)
C23—C25—C26—C27	−178.5 (3)	C21—C20—N5—C22	−137.7 (3)
C25—C26—C27—C28	−0.3 (5)	O9—C22—N6—C23	−0.4 (5)
C26—C27—C28—O12	178.8 (3)	N5—C22—N6—C23	−178.9 (2)
C26—C27—C28—C29	−0.4 (5)	C25—C23—N6—C22	170.7 (2)
O12—C28—C29—C30	−178.6 (3)	C24—C23—N6—C22	−68.5 (3)
C27—C28—C29—C30	0.6 (5)	O11—C24—O10—O5	−2.3 (3)
C28—C29—C30—C25	−0.1 (5)	C23—C24—O10—O5	174.5 (3)
C26—C25—C30—C29	−0.5 (5)	C8—O5—O10—C24	162.7 (3)
C23—C25—C30—C29	178.7 (3)		

### Hydrogen-bond geometry (Å, °)

**Table:**

*D*—H···*A*	*D*—H	H···*A*	*D*···*A*	*D*—H···*A*
O11—H11O···O5	0.86 (3)	1.80 (4)	2.617 (3)	159 (9)
O10—H10O···O4	0.86 (3)	1.82 (3)	2.678 (3)	171 (9)
O5—H5O···O11	0.86 (3)	1.76 (3)	2.617 (3)	171 (8)
O4—H4O···O10	0.86 (3)	1.88 (5)	2.678 (3)	154 (9)
N6—H6D···O8	0.86	1.98	2.637 (3)	132
N3—H3···O2	0.86	1.97	2.636 (3)	133
O6—H6···O1^i^	0.82	1.87	2.682 (3)	174
C12—H12···O2^i^	0.93	2.52	3.438 (4)	169
O12—H12A···O7^ii^	0.82	1.88	2.692 (3)	172
C27—H27···O8^ii^	0.93	2.50	3.378 (4)	158

Symmetry codes: (i) *x*+1/2, −*y*+1/2, −*z*+1; (ii) *x*+1/2, −*y*+5/2, −*z*+1.
